# Supplementary material for: Fragile protein folds: sequence and environmental factors affecting the equilibrium of two interconverting, stably folded protein conformations
Source: Magn Reson (Gott). 2021 Mar 10;2(1):63–76. doi: 10.5194/mr-2-63-2021 (PMC9119131; doi:10.5194/mr-2-63-2021)
Supplement: The Supplement contains Figs. S1–S9. The supplement related to this article is available online at: https://doi.org/10.5194/mr-2-63-2021-supplement. [file mr-2-63-supplement.pdf]

Supplement of Magn. Reson., 2, 63–76, 2021
https://doi.org/10.5194/mr-2-63-2021-supplement
© Author(s) 2021. This work is distributed under
the Creative Commons Attribution 4.0 License.

Open Access
MAGNETIC
RESONANCE

Supplement of

Fragile protein folds: sequence and environmental factors affecting the equilibrium of two interconverting, stably folded protein conformations

Xingjian Xu et al.

Correspondence to: Kevin H. Gardner (kgardner@gc.cuny.edu)

The copyright of individual parts of the supplement might differ from the CC BY 4.0 License.

Supplementary Figures

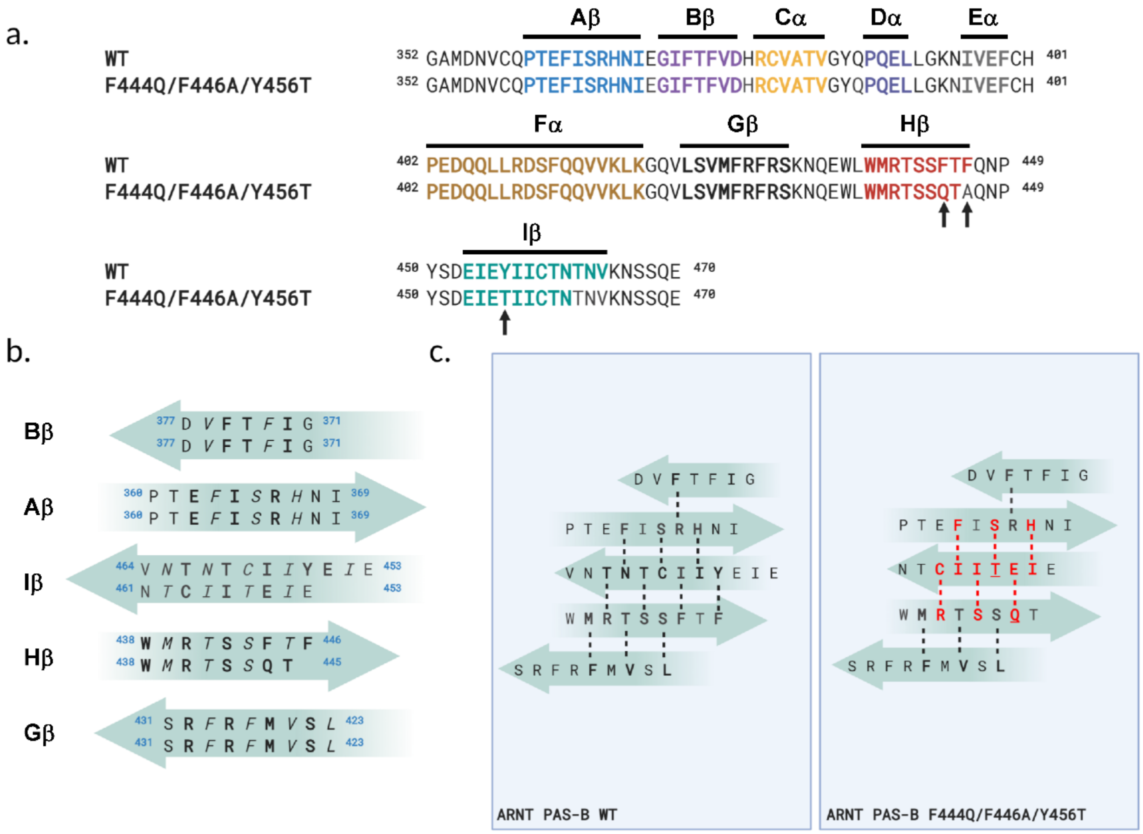

Supplementary Figure S1 – Sequence comparison and changes in backbone hydrogen bonding between WT and SLIP conformations. (a) Sequence alignment (EMBOSS stretcher (Madeira et al., 2019)) of WT (WT conformation) and F444Q/F446A/Y456T (SLIP conformation-locked) variant, with locations of key secondary structures labeled. (b) Comparison of the five antiparallel β-strands between WT and F444Q/F446A/Y456T variant. External-orienting residues (bold) and internal-orienting residues (italic) are marked. Some residues on the two ends of strands are not labeled as they are transitioning into connecting loops. The Iβ-strand of the mutant shows a 3-residue slip and an inversion of topology. (c) Backbone H-bonds between β-strands are shown for both WT and F444Q/F446A/Y456T variant. Changes are highlighted in the panel showing the H-bonds of the mutant (red).

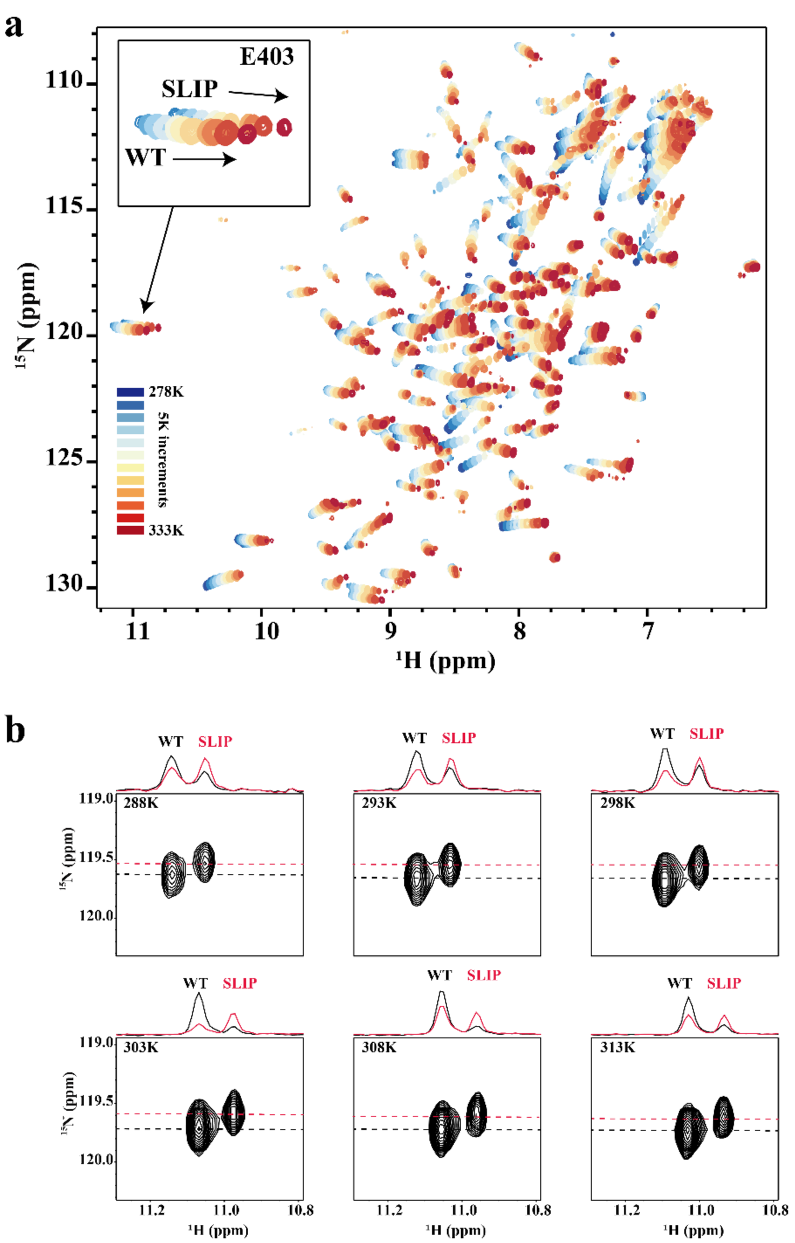

Supplementary Figure S2 – Temperature dependence of the WT:SLIP conformational equilibrium in ARNT PAS-B Y456T. (a) Variable temperature ^{15}N/^{1}H HSQC spectra of ARNT PAS-B Y456T, recorded in 5 degree increments between 278K and 333K. Inset shows the temperature-dependent chemical shift changes of a pair of peaks representing the two conformations adopted by residue E403. (b) Expansion of the E403 signal in ^{15}N/^{1}H HSQC spectra at indicated temperatures (288K – 313K). Relative populations are indicated on the edge of each figure, showing intensities of slices through the center of the WT peak (black) and the SLIP peak (red).

a.

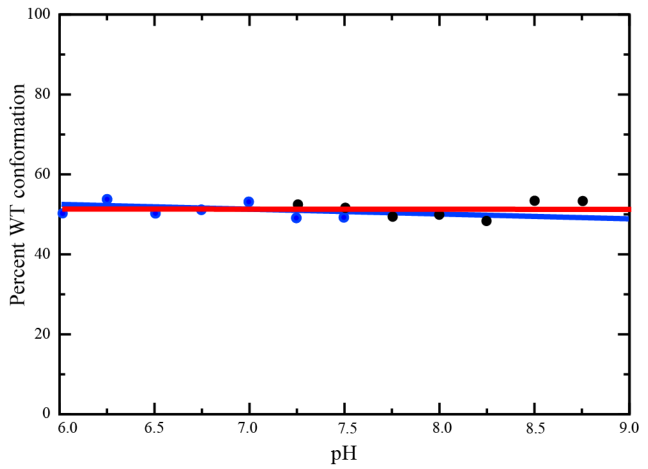

b.

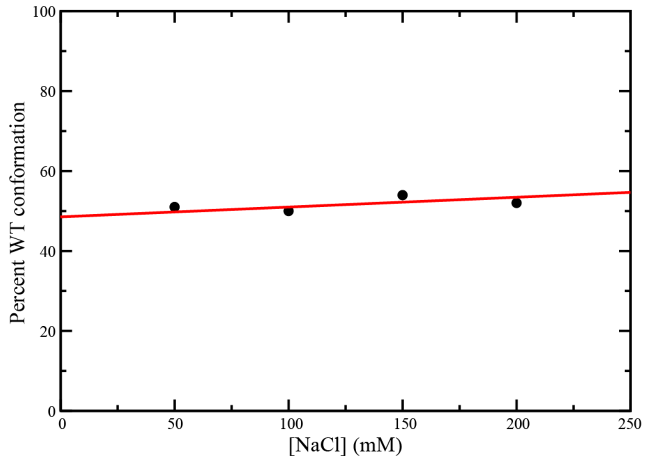

Supplementary Figure S3 – pH and salt concentration have negligible effects on WT:SLIP equilibrium in ARNT PAS-B Y456T. (a) The WT:SLIP equilibrium remains unchanged between pH 7.0 and 9.0 in Tris (black circles, red line) or between pH 6.0 and 7.5 in PIPES (blue circles, blue line), with 17 mM NaCl present at all points. The lines represent linear regressions to the data points. (b) The WT:SLIP equilibrium is chiefly unchanged throughout a NaCl titration between 50 and 200 mM (black circles, red line), all at pH 7.5. The line represents a linear regression to the data points.

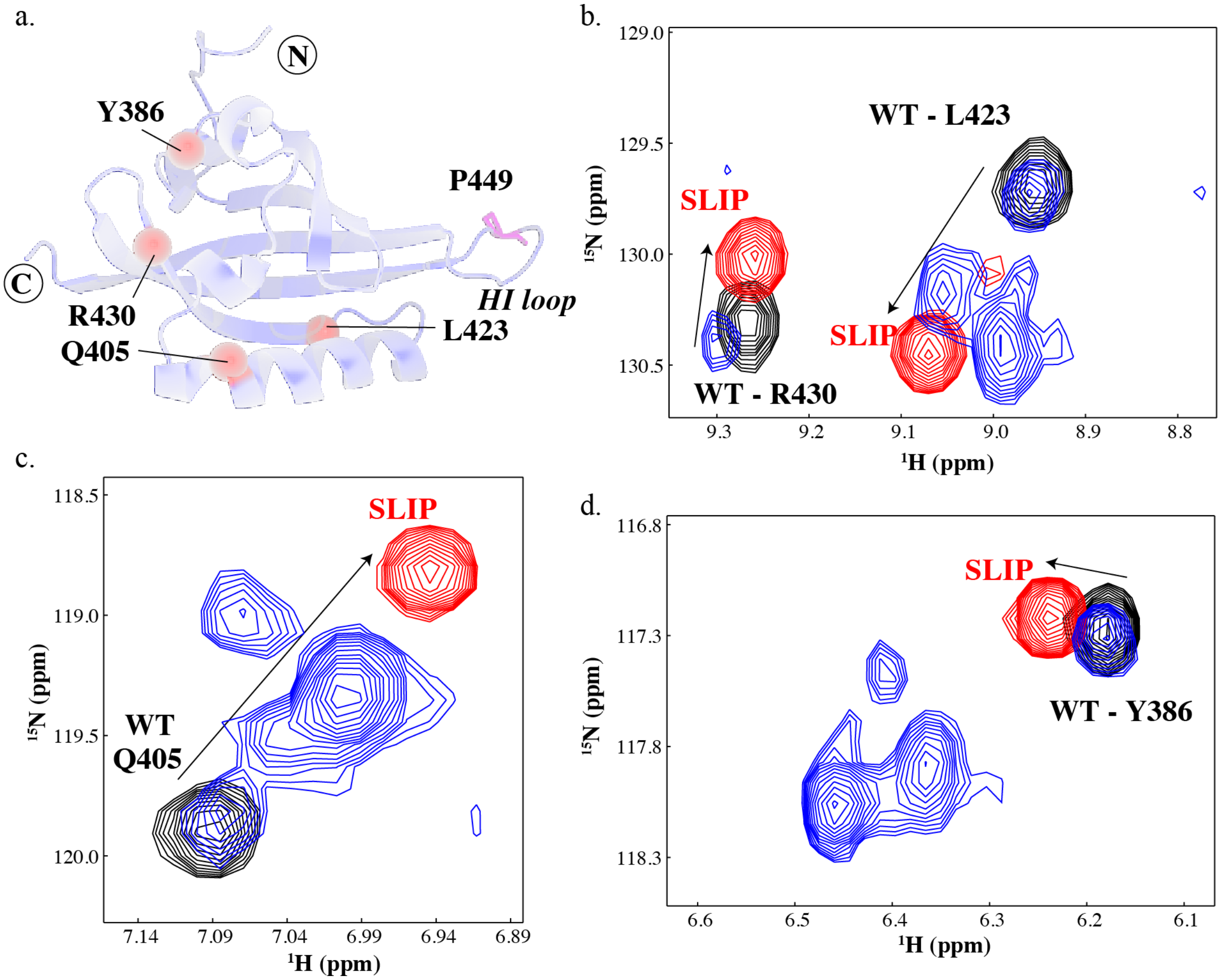

Supplementary Figure S4 – P449A point mutation generates multiple conformations of ARNT PAS-B. (a) A schematic of the solution structure of wildtype ARNT PAS-B domain (PDB:1X0O (Card et al., 2005)), highlighting the location of the P449 residue (magenta sticks) and residues indicated in panels b-d. (b-d) Widespread peak multiplicity in the P449A mutant is observed throughout ^{15}N/^{1}H HSQC spectra as probed at residues Y386, Q405, L423, and R430. Four major species are observed: One at the WT location (14% intensity), and three additional ones at locations which do not overlap the SLIP location. Approximate populations of these peaks are 60%, 14%, and 12%; assignments were made by assuming that the largest peak for each residue corresponded to the dominant (60%) state, the next state corresponded to the peak closest to the WT-SLIP vector, followed by peaks further from this vector. Proteins: wildtype (black), F444Q/F446A/Y456T (red) and P449A (blue).

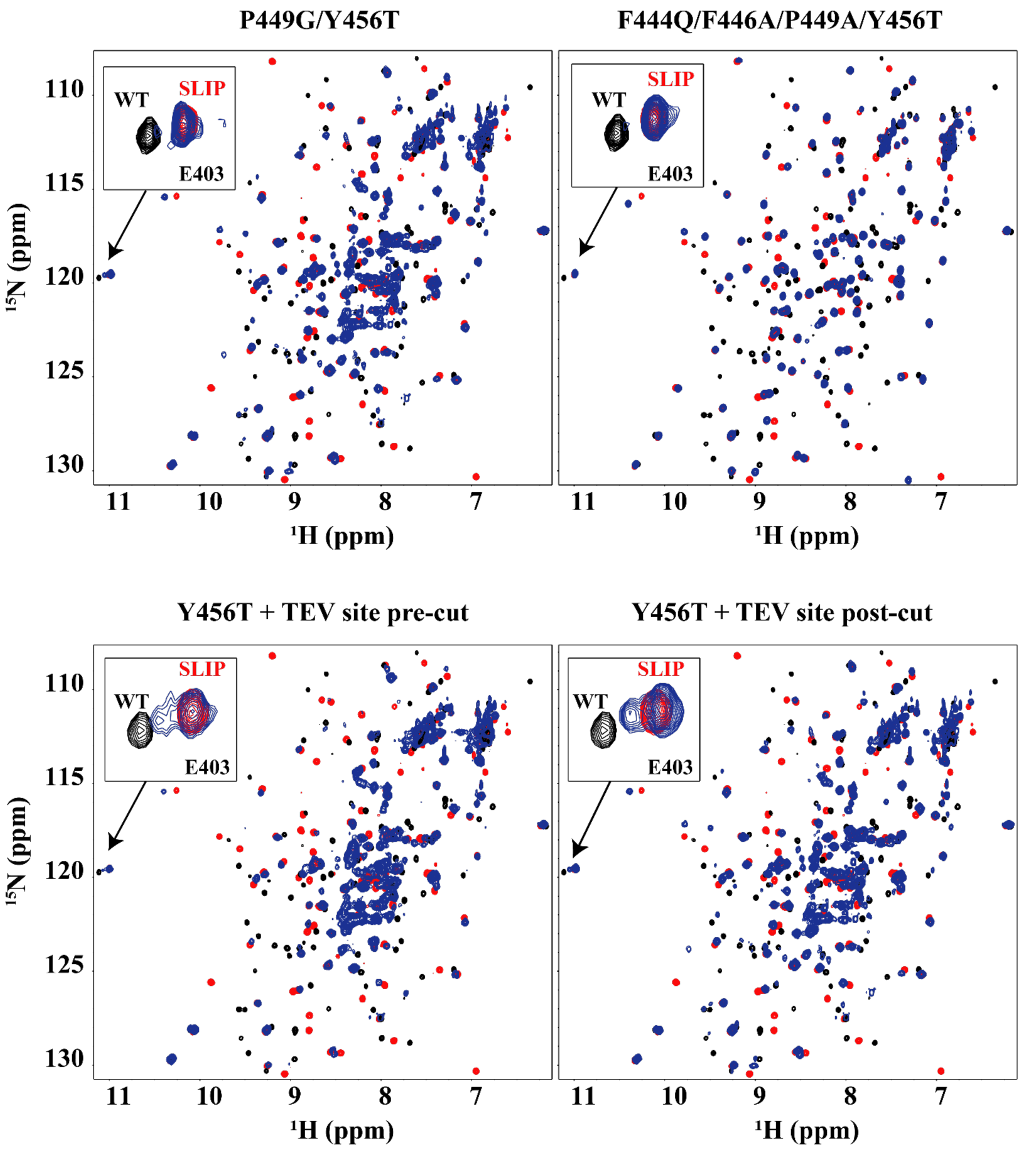

Supplementary Figure S5 – ^{15}N/^{1}H HSQC spectra of ARNT PAS-B sequence variants presented in Table 1. Spectra of ARNT PAS-B variants P449G/Y456T, F444Q/F446A/P449A/Y456T, and Y456T + TEV (pre- and post-cleavage) are shown (blue). ^{15}N/^{1}H HSQC spectra of WT (black) and F444Q/F446A/Y456T (red) are also shown as reference. Insets show the relative population of residue E403 in each variant.

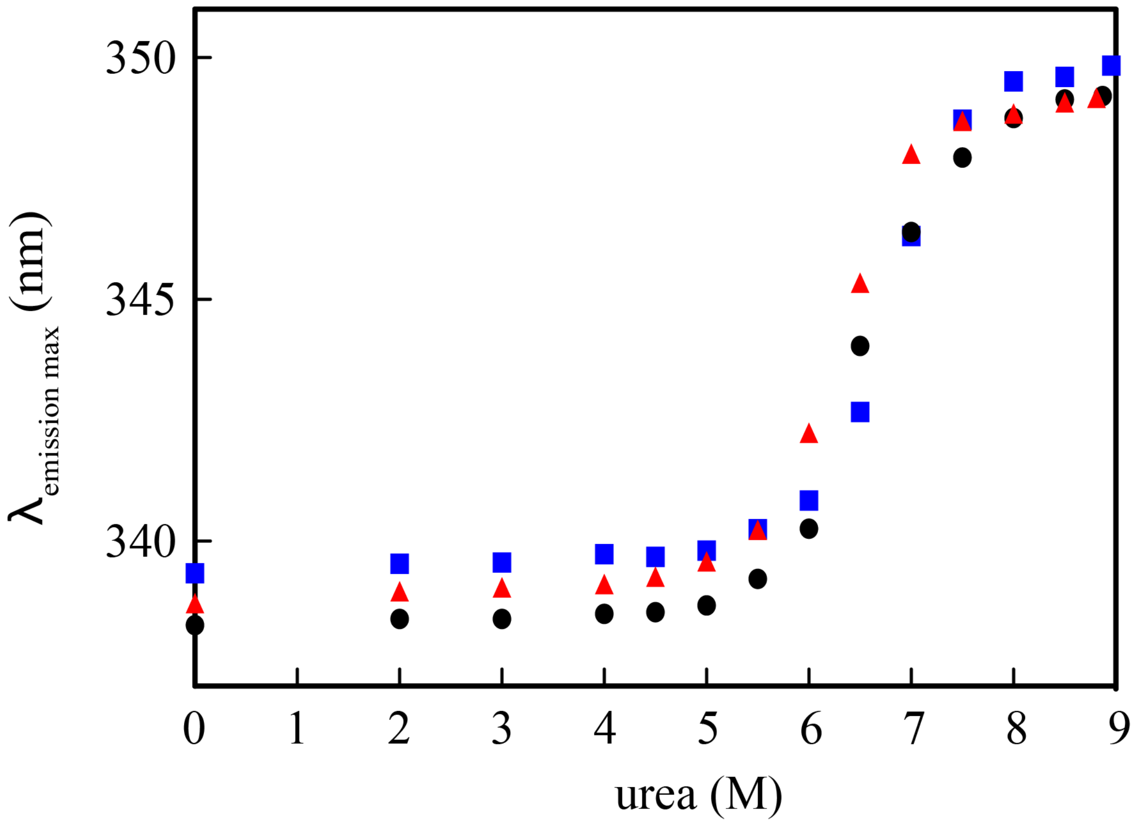

Supplementary Figure S6 – ARNT PAS-B variants have similar stabilities as probed by Trp fluorescence during urea denaturation. The peak emission wavelength of ARNT PAS-B wildtype (black circles), Y456T (red triangles), and F444A/F446Q/Y456T (blue squares) red shifted with increasing concentrations of urea as the proteins unfolded. Comparable stabilities of all three variants were observed.

KG-279

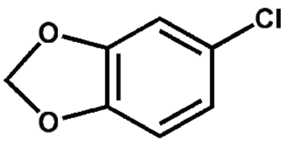

WT + 500 μM KG279

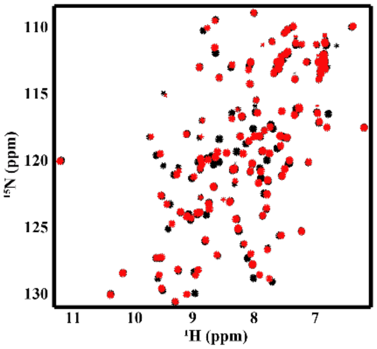

TRIP + 500 μM KG279

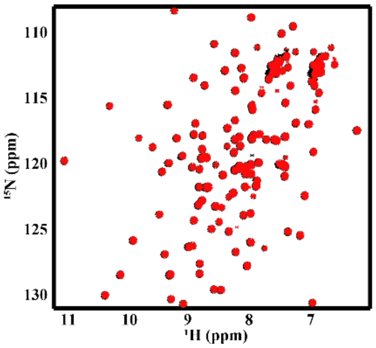

KG-499

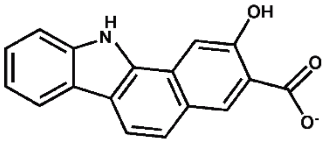

WT + 500 μM KG499

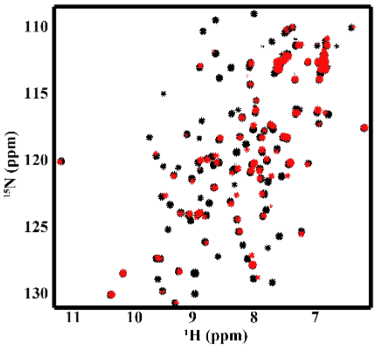

TRIP + 500 μM KG499

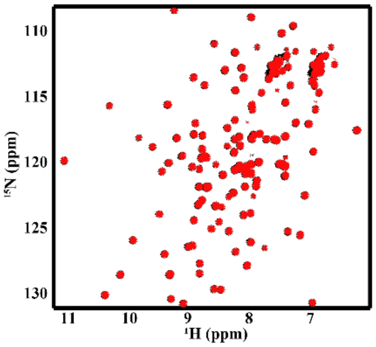

KG-547

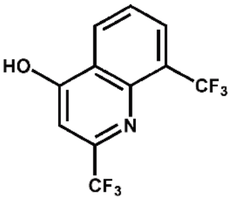

WT + 500 μM KG547

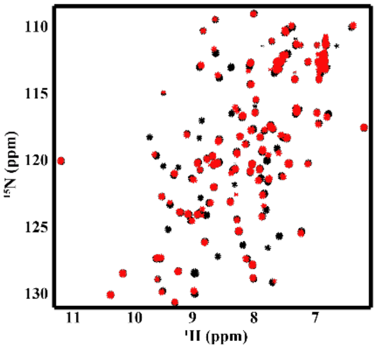

TRIP + 500 μM KG547

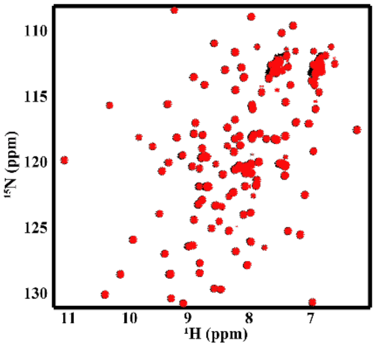

KG-580

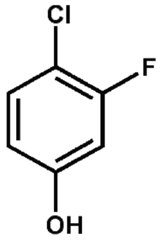

WT + 500 μM KG580

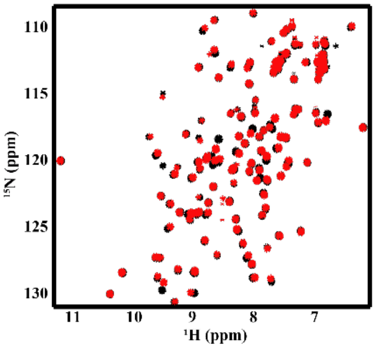

TRIP + 500 μM KG580

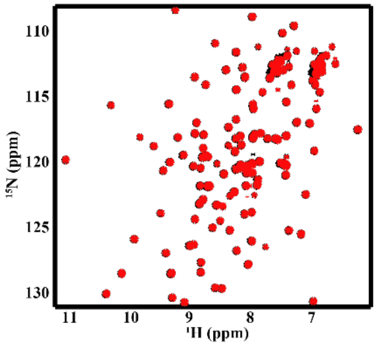

KG-586

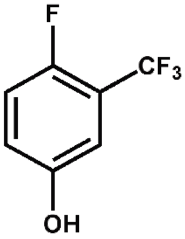

WT + 500 μM KG586

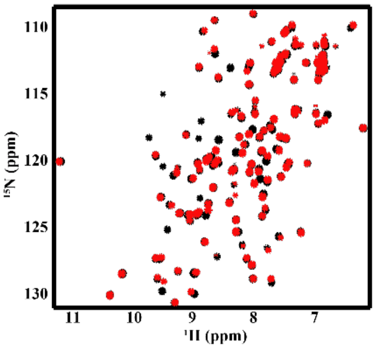

TRIP + 500 μM KG586

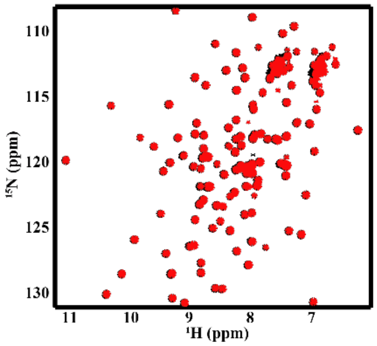

KG-630

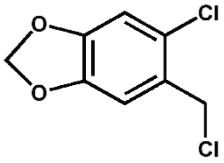

WT + 500 μM KG630

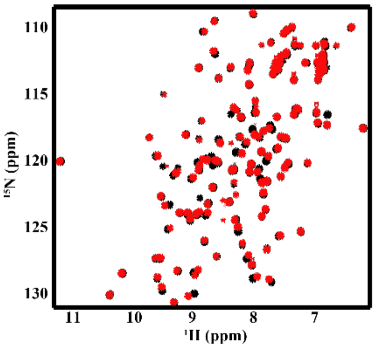

TRIP + 500 μM KG630

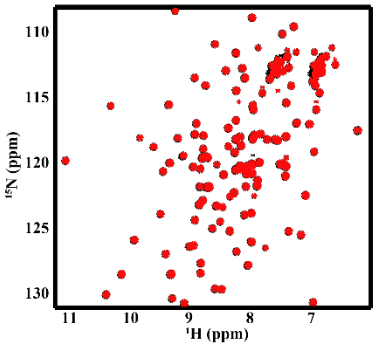

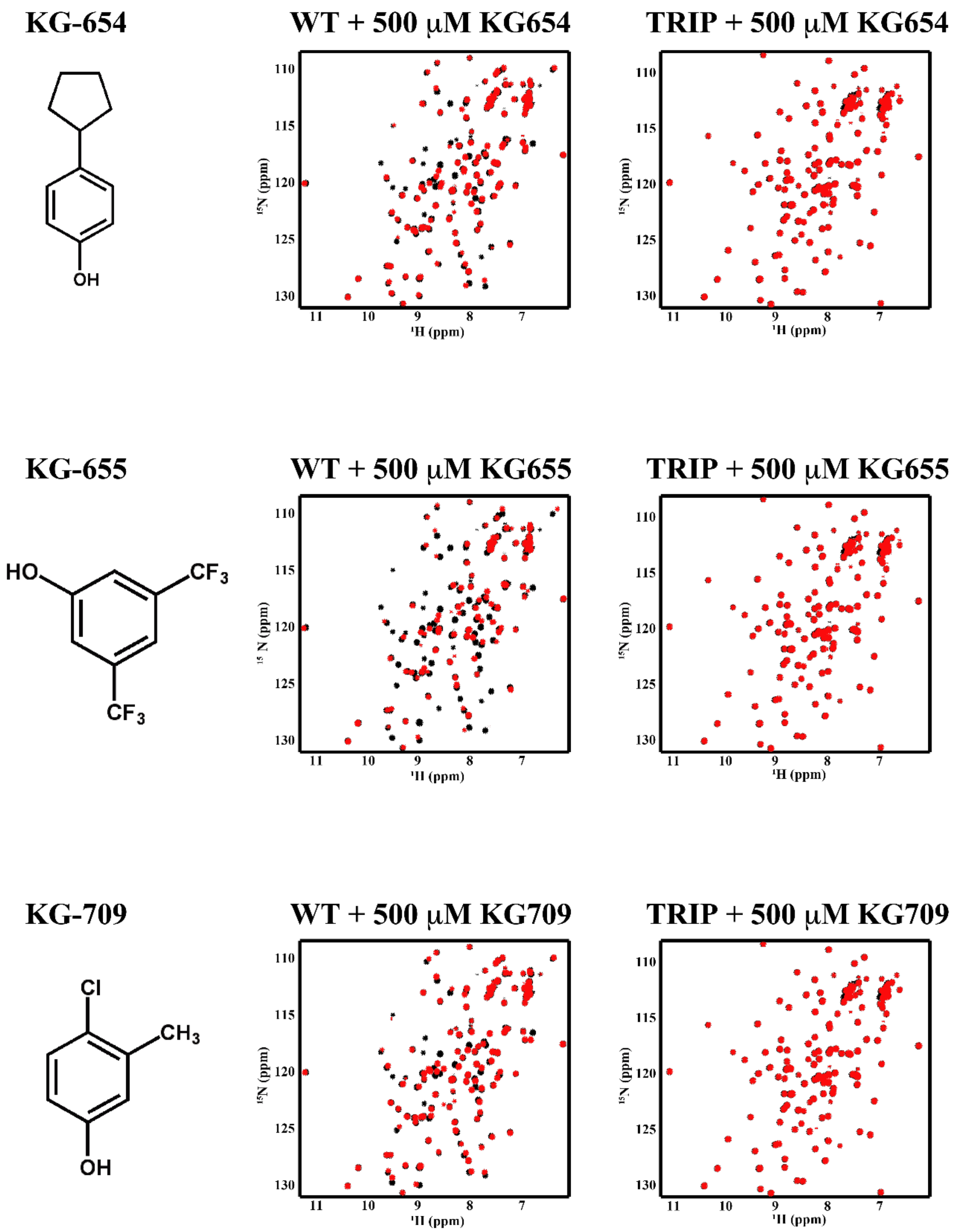

Supplementary Figure S7 – Counter-screen of ligands that bind to F444Q/F446A/Y456T triple mutant (TRIP). Ligands that bind to ARNT PAS-B WT (Guo et al., 2013) were tested for binding against the TRIP mutant. Shown are $^{15}N/^{1}H$ HSQC spectra of WT ARNT PAS-B and TRIP mutant without (black) or with 500 μM of ligands added (red) as indicated, in the presence of 2% DMSO.

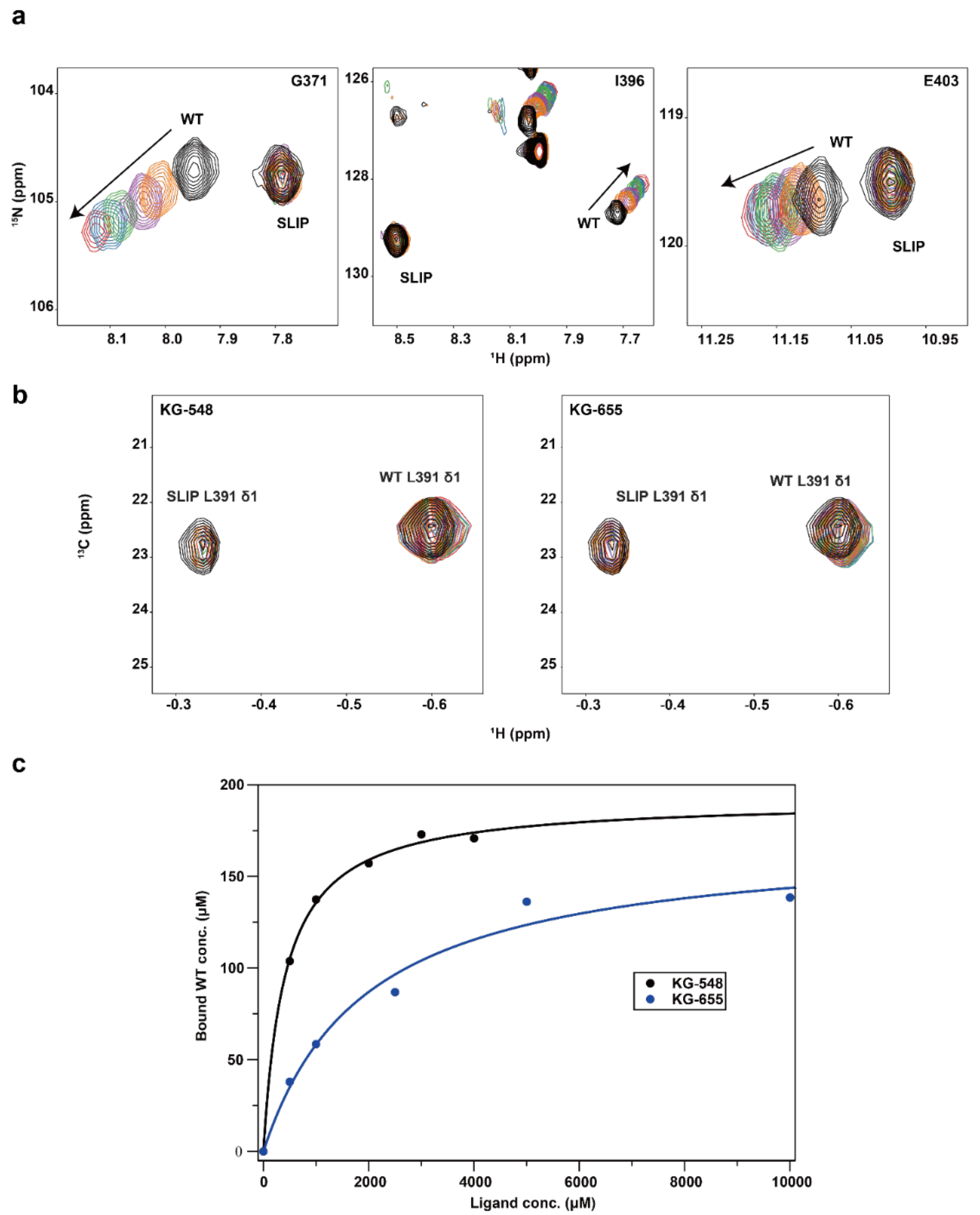

Supplementary Figure S8 – KG-548 and KG-655 binding titration analysis using $^{13}C/^{1}H$ HSQC. (a) Example peaks from $^{15}N/^{1}H$ HSQC spectra of KG-655 titration series (0, 500, 1000, 2500, 5000, 10000 μM) against 250 μM ARNT PAS-B Y456T. Peaks associated with the WT conformation show chemical shift changes, while peaks associated with the SLIP conformation do not, indicating selective binding. Unlike KG-548, many analyzable residues (where both WT and SLIP peaks are assigned) are directly involved with ligand binding and showing mixed fast-intermediate exchange characteristics in NMR spectra. (b) $^{13}C/^{1}H$ HSQC titration experiments of KG-548 (0, 500, 1000, 2000, 3000, 4000 μM) and KG-655 (0, 500, 1000, 2500, 5000, 10000 μM) against 250 μM ARNT PAS-B Y456T, focusing on the L391

δ1 methyl signals. Both WT and SLIP peaks are well resolved at all compound concentrations with no sign of peak broadening. Chemical shift changes to the WT conformation are minimal in the presence of both KG-548 and KG-655, suggesting the residue is not directly involved in the binding of either compounds. (c) KG-548 and KG-655 binding to ARNT PAS-B Y456T as monitored by peak volumes of L391 δ1 from $^{13}C/^{1}H$ HSQC NMR spectra. Data are fit to Eq. 1 to extract dissociation constant K_d and maximum binding B_{max}; resulting values are $K_d = 414 \pm 7.1$ μM and $B_{max} = 192 \pm 0.96$ μM for KG-548, and $K_d = 1947 \pm 152$ μM and $B_{max} = 172 \pm 5.7$ μM for KG-655. Uncertainties were estimated using bootstrapping. Noises with mean of 0 and variance of the standard error were added to the experimental data. Generated datasets (n = 30) were fit to obtain the 95% confidence interval.

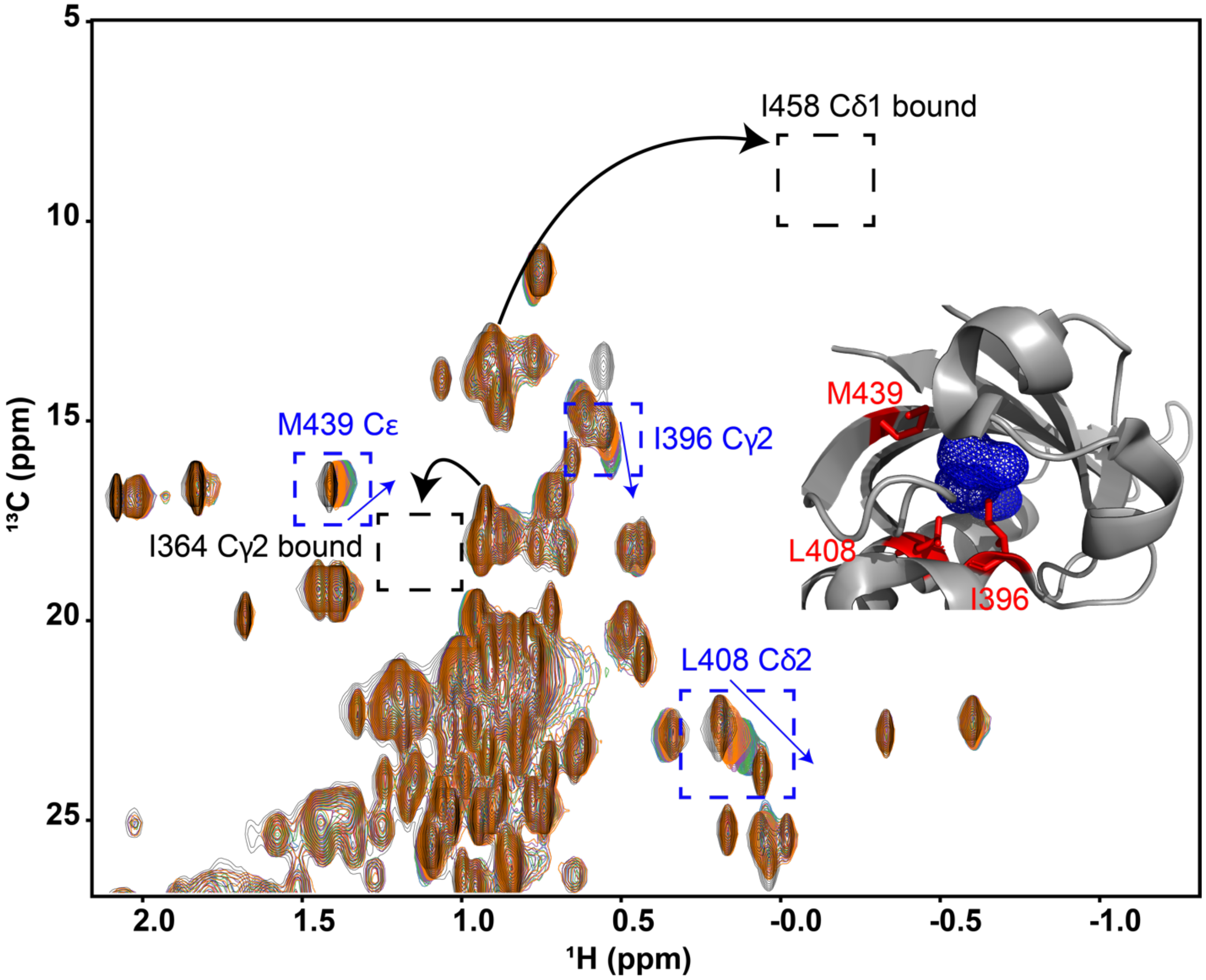

Supplementary Figure S9 – KG-655 loses surface binding to the WT conformation of ARNT PAS-B Y456T while retaining the internal binding mode. Shown here is the $^{13}C/^{1}H$ HSQC titration series of KG-655 (0 – 10 mM) against ARNT PAS-B Y456T, zoomed in on the methyl region. As previously reported, KG-655 binds to wildtype ARNT PAS-B via two binding modes (Gagné, 2020). The surface binding of KG-655 involves sidechains of residue I364 and I458, resulting in chemical shift changes that are observed when titrated against wildtype ARNT PAS-B, but are missing from these spectra even at the highest ligand concentration (black dotted squares). Methyl groups with changes in chemical shifts (I396, L408, and M439, blue dotted squares) are all on sidechains oriented inward (inset PDB:4EQ1 (Guo et al., 2013)), to the internal cavity of the protein, suggesting the internal binding mode is retained.

References

Card, P. B., Erbel, P. J. A., and Gardner, K. H.: Structural basis of ARNT PAS-B dimerization: use of a common beta-sheet interface for hetero- and homodimerization, J. Mol. Biol., 353, 664-677, 10.1016/j.jmb.2005.08.043, 2005.

Gagné, D. Azad, R.; Edupuganti, U.R.; Williams, J.; Aramini, J.M.; Akasaka, K., and Gardner, K.H.: Use of high pressure NMR spectroscopy to rapidly identify internal ligand-binding voids in proteins, preprint on bioRxiv, 10.1101/2020.08.25.267195, 2020.

Guo, Y., Partch, C. L., Key, J., Card, P. B., Pashkov, V., Patel, A., Bruick, R. K., Wurdak, H., and Gardner, K. H.: Regulating the ARNT/TACC3 axis: multiple approaches to manipulating protein/protein interactions with small molecules, ACS Chem. Biol., 8, 626-635, 10.1021/cb300604u, 2013.

Madeira, F., Park, Y. M., Lee, J., Buso, N., Gur, T., Madhusoodanan, N., Basutkar, P., Tivey, A. R. N., Potter, S. C., Finn, R. D., and Lopez, R.: The EMBL-EBI search and sequence analysis tools APIs in 2019, Nucleic Acids Res., 47, W636-W641, 10.1093/nar/gkz268, 2019.
